# Channel *HCN4* mutation R666Q associated with sporadic arrhythmia decreases channel electrophysiological function and increases protein degradation

**DOI:** 10.1016/j.jbc.2022.102599

**Published:** 2022-10-14

**Authors:** Hongrui Wang, Tong Wu, Zhuo Huang, Jinghan Huang, Ze Geng, Bing Cui, Yupeng Yan, Yu Zhang, Yibo Wang

**Affiliations:** 1 State Key Laboratory of Cardiovascular Disease, Fuwai Hospital, National Center for Cardiovascular Diseases, Chinese Academy of Medical Sciences and Peking Union Medical College, Beijing, China; 2 State Key Laboratory of Natural and Biomimetic Drugs, Department of Molecular and Cellular Pharmacology, School of Pharmaceutical Sciences, Peking University Health Science Center, Beijing, China; 3 Functional Testing Center, Fuwai Hospital, Chinese Academy of Medical Sciences and Peking Union Medical College, Beijing, China

**Keywords:** HCN4, bradycardia, QT prolongation, short bursts of ventricular tachycardia, electrophysiology, bpm, beats per minute, CHX, cycloheximide, HCN, hyperpolarization-activated cyclic nucleotide-gated, CNBD, cyclic nucleotide-binding domain, UPS, ubiquitin-proteasome system, SUMO, small ubiquitin-related modifier

## Abstract

Mutations in the hyperpolarization-activated nucleotide-gated channel 4 (HCN4) are known to be associated with arrhythmias in which QT prolongation (delayed ventricular repolarization) is rare. Here, we identified a *HCN4* mutation, *HCN4*-R666Q, in two sporadic arrhythmia patients with sinus bradycardia, QT prolongation, and short bursts of ventricular tachycardia. To determine the functional effect of the mutation, we conducted clinical, genetic, and functional analyses using whole-cell voltage-clamp, qPCR, Western blot, confocal microscopy, and co-immunoprecipitation. The mean current density of HEK293T cells transfected with *HCN4*-R666Q was lower in 24 to 36 h after transfection and was much lower in 36 to 48 h after transfection relative to cells transfected with wildtype HCN4. Additionally, we determined that the HCN4-R666Q mutant was more susceptible to ubiquitin-proteasome system–mediated protein degradation than wildtype HCN4. This decreased current density for HCN4-R666Q could be partly rescued by treatment with a proteasome inhibitor. Therefore, we conclude that HCN4-R666Q had an effect on HCN4 function in two aspects, including decreasing the current density of the channel as a biophysical effect and weakening its protein stability. Our findings provide new insights into the pathogenesis of the *HCN4*-R666Q mutation.

Sinus bradycardia is defined as a heart rate less than 60 beats per minute (bpm) in adults (1). Asymptomatic sinus bradycardia is usually harmless and is often a sign of good physical conditioning, whereas symptomatic sinus bradycardia is associated with sick-sinus syndrome and can be a life-threatening condition (1). Accordingly, permanent pacing is required for the majority of patients with symptomatic sinus bradycardia (2, 3, 4).

Pacemaker current (*I*_f_) carried by the hyperpolarization-activated cyclic nucleotide-gated (HCN) cation channel plays a specific role in pacing and heart rate control (5). Of the four isoforms encoding HCN channels (HCN1-4), HCN4 is the most abundantly expressed in the mammalian sinoatrial node and underlies the I_f_ (6, 7, 8, 9, 10). HCN4 has six transmembrane helices (S1–S6) and a cyclic nucleotide-binding domain (CNBD) in the middle of the C terminus (11, 12). HCN4 channel is directly regulated by cAMP, which binds to three binding sites (residues 659–662, 669–670 and 710–713) on CNBD and elicits a positive shift in the voltage dependence of activation (9, 13). Several studies have shown that a wide variety of mutations of HCN channels are involved in the pathogenesis of inherited sinus node dysfunction by altering channel biophysical properties, including *HCN*4-695X, S672R, D553N, G482R, A485V, G480R, 573X, G811E, I479V, and A485E (1, 9, 14, 15, 16, 17, 18, 19, 20, 21, 22, 23, 24). *HCN4* mutations is associated with arrhythmias including Brugada syndrome (*HCN*4-V492F) (25), left ventricular noncompaction (*HCN*4-G482R, G811E) (16, 21), atrioventricular block (*HCN*4-G1097W) (11), and atrial fibrillation (*HCN*4-P257S, K530N) (8, 26). Newborn infants with QT prolongation often show sinus bradycardia (27). Almost all of the ion channels affected by *LQTS* gene mutations are expressed in the human sinoatrial node, and it is the same with HCN4 channel (6, 28). Therefore, LQTS is frequently associated with a change in basal heart rate due to impaired SAN pacemaker activity (29). Although sinus bradycardia has been reported in relation to LQTS or HCN4 mutations, mutations in HCN4 are known to be associated with arrhythmias in which QT prolongation is rare. These changes mimic the effect of vagal stimulation. Known *HCN4* mutations are almost all located in the C-linker (9, 19, 30), pore region (16, 17, 18), or C-terminal domains (8, 11, 21).

Here, we identified a novel heterozygous mutation *HCN4*-R666Q, which was located in the non-cAMP-binding region of CNBD, in two sporadic patients with sinus bradycardia, QT prolongation, and short bursts of ventricular tachycardia. We hypothesized that *HCN4*-R666Q mutation was associated with the clinical phenotype of two sporadic patients and explored the mechanism of channel dysfunction caused by the mutation.

## Results

### HCN4 mutation R666Q was found in two sporadic patients with sinus bradycardia, QT prolongation, and short bursts of ventricular tachycardia

**Figure 1:**
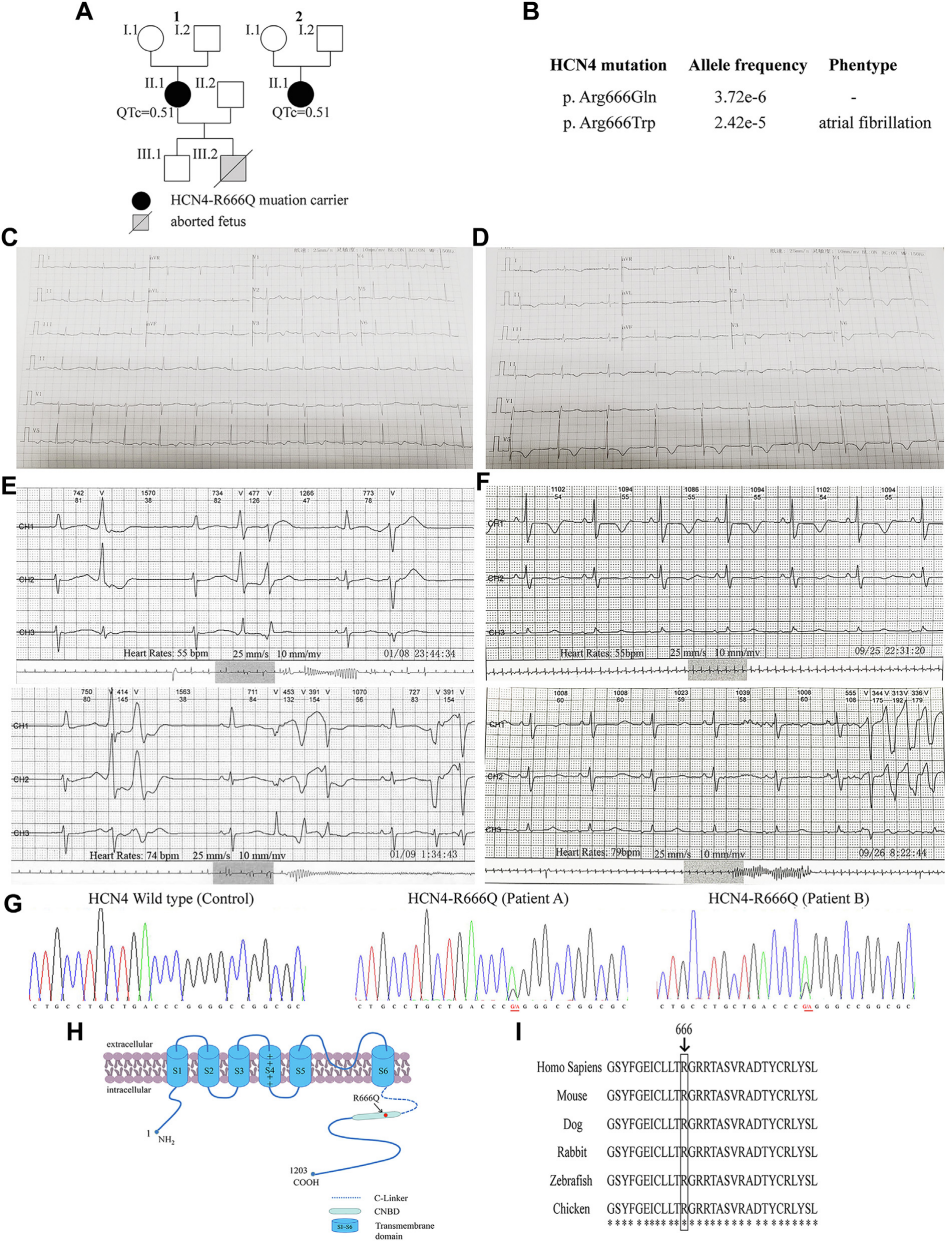
**Genetic identification of the *HCN4*-R666Q mutation**. *A*, pedigrees of the two unrelated carriers of the mutation. *Closed symbols* indicate affected, *open symbols* denote unaffected family members, and *gray symbols* indicate an unknown phenotype. *Circles* refer to women, *squares* indicate men. *Slashed symbols* indicate deceased subjects. The QTc of each affected individual is noted. *B*, allele frequency of *HCN4*-R666 mutation in UK biobank. *C*, 12-lead ECGs of patient A. *D*, 12-lead ECGs of patient B. *E*, 24-h Holter monitoring of one patient (40 years) showed sinus bradycardia (average heart rate <60 bpm) and QT prolongation (QT-intervals = 0.52 s; QTc = 0.51 s) and concurrent with short bursts of ventricular tachycardia and accompanied with premature ventricular complexes. *F*, 24-h Holter monitoring of the other patient (45 years) showed sinus bradycardia (average heart rate <60 bpm) and QT prolongation (QT-intervals = 0.48 s; QTc = 0.51 s) and concurrent with short bursts of ventricular tachycardia. *G*, Sanger sequence analysis of wildtype (*left*) and HCN4-R666Q mutant DNA (*right*) found in two sporadic patients revealed a transition of nucleotide G1997 to A. *H*, predicted HCN4 subunit topology. The *HCN4*-R666Q mutation was indicated by *arrow*. S = transmembrane segment. *I*, multiple alignment of the HCN4-R666, showed the conserved arginine in position 666 in six different species (*Homo* sapiens, dog, rabbit, zebrafish, chicken, mouse).

Two female patients (40 years; 1-II.1 and 45 years; 2-II.1; Fig. 1*A*) suffering from palpitation and syncope were admitted to Fuwai Hospital as outpatients. On arrival, the heart rate of two patients was slowed down (patient A: 46 bpm, patient B: 54 bpm), but their blood pressure was normal (patient A: 115/88 mm Hg, patient B: 110/80 mm Hg). According to the two patients’ descriptions, they were the only child in their families. Patient A had a son and an aborted fetus, while patient B had no children. Panel sequencing showed that the two patients with clinical manifestations of sinus bradycardia, QT prolongation, and short bursts of ventricular tachycardia carried *HCN4*-R666Q (c.1997G > A, NM_005477) mutation in which glutamine was substituted for arginine. In addition, no other significant mutations in arrhythmia-related genes were observed in the two patients (Table 1). Analysis of UK biobank (www.https://genebass.org/) suggested that the HCN4 p. Arg666Trp mutation was associated with susceptibility to atrial fibrillation (*p* = 9.7e-4), indicating the potential significance of this residue in HCN4 (Fig. 1*B*). Holter monitoring showed the two patients have lower average (patient A: 48 bpm, patient B: 52 bpm) and minimum (patient A: 44 bpm, patient B: 46 bpm) heart rates. 24-h Holter recordings were analyzed by a computer system and confirmed by an electrophysiologist. The patients were diagnosed as having sinus bradycardia with average heart rate <60 bpm concurrent with QT prolongation with QT-intervals >0.44 s (Patient A: QT-intervals = 0.52 s; the corrected QT (QTc) corrected QT-intervals = 0.51 s; Patient B: QT-intervals = 0.48 s; QTc = 0.51 s) accompanied by short bursts of ventricular tachycardia as assessed by 24-h Holter monitoring and 12-lead ECG (Fig. 1, *C*–*F*). Patient A had premature ventricular complexes (Fig. 1*E*) and two patients had no history of coronary heart disease or drug-induced QT prolongation. No abnormal changes were observed in plasma ion concentrations of two patients, including sodium, potassium, magnesium, and calcium. Echocardiography examination of two patients showed no structural heart disease (Table S1). DNA sequencing, 12-lead ECG, and Doppler echocardiography results for the two patients’ parents and patient A’s son showed no mutation or structural heart disease were observed (Figs. S1 and S2 and Table S1).

**Table 1:** List of arrhythmia-related genes analyzed in the panel sequencing

Gene	Protein
*KCNQ1 (LQT1)*	Potassium channel alpha subunit (KvLQT1,Kv7.1)
*KCNH2 (LQT2)*	Potassium channel α subunit (HERG, Kv11.1)
*SCN5A (LQT3)*	Voltage-gated sodium channel α subunit (Nav1.5)
*AKAP9 (LQT11)*	Yotiao
*CACNA1C (LQT8)*	Voltage gated L-type calcium channel alpha 1 subunit (Cav1.2)
*CALM1 (LQT14)*	Calmodulin
*CAV3 (LQT9)*	Caveolin-3
*KCNE1 (LQT5)*	Kv7.1 potassium channel beta subunit (MinK)
*KCNE2 (LQT5/6)*	Kv11.1 potassium channel beta subunit (MiRP1)
*KCNE3*	Potassium channel beta subunit (MiRP2)
*KCNJ2 (LQT7)*	IK1 potassium channel (Kir2.1)
*KCNJ5 (LQT13)*	Potassium inwardly-rectifying channel (Kir3.4)
*KCNJ8 (LQT8)*	Potassium inwardly-rectifying channel (Kir6.1)
*ANK2 (LQT4)*	Ankyrin B
*DSP*	Desmoplakin
*DSG2*	Desmoglein-2
*DSC2*	Desmocollin-2
*SCN4B*	Sodium channel beta 4 subunit
*SNTA1*	Syntrophin-alpha 1
*CACNB2*	Voltage-dependent L-type calcium channel subunit beta-2
*PKP2*	Plakophilin-2
*RANGRF*	Ran guanine nucleotide release factor
*TMEM43*	Transmembrane protein 43
*RYR2*	Ryanodine receptor 2
*TRDN*	Triadin
*GPD1L*	Glycerol-3-phosphate dehydrogenase 1-like protein
*CACNB2*	Voltage-dependent L-type calcium channel subunit beta-2
*SCN1B*	Sodium channel subunit beta-1
*SCN3B*	Sodium channel subunit beta-3
*HCN4*	Hyperpolarization-activated cyclic nucleotide-gated channel 4
*CASQ2*	Calsequestrin-2
*JUP*	Junction plakoglobin

### In silico analysis showed the mutation was pathogenic and evolutionarily conserved

Sanger sequencing (Fig. 1*G*) showed that arginine 666 was replaced with a glutamine (R666Q) in the highly conserved CNBD of the HCN4 subunit (Fig. 1*H*). This mutation was not found among the 1000 genomes and EPS6500 database, and no minor allele frequency was observed in the general population in the Exome Aggregation Consortium database (21). Furthermore, the *HCN4*-R666Q mutation was predicted to have a high pathogenicity index in *silico* analysis with Mutation Taster (disease causing), PROVEAN (deleterious), FATHMM (damaging), and PolyPhen-2 (possibly damaging). A multiple alignment (ClustalX2.0.10) showed that arginine 666 was conserved across a wide range of mammals, including *Homo* sapiens, dog, rabbit, zebrafish, chicken, and mouse (Fig. 1*I*).

### Reduced current density of HCN4-R666Q mutant channels

**Figure 2:**
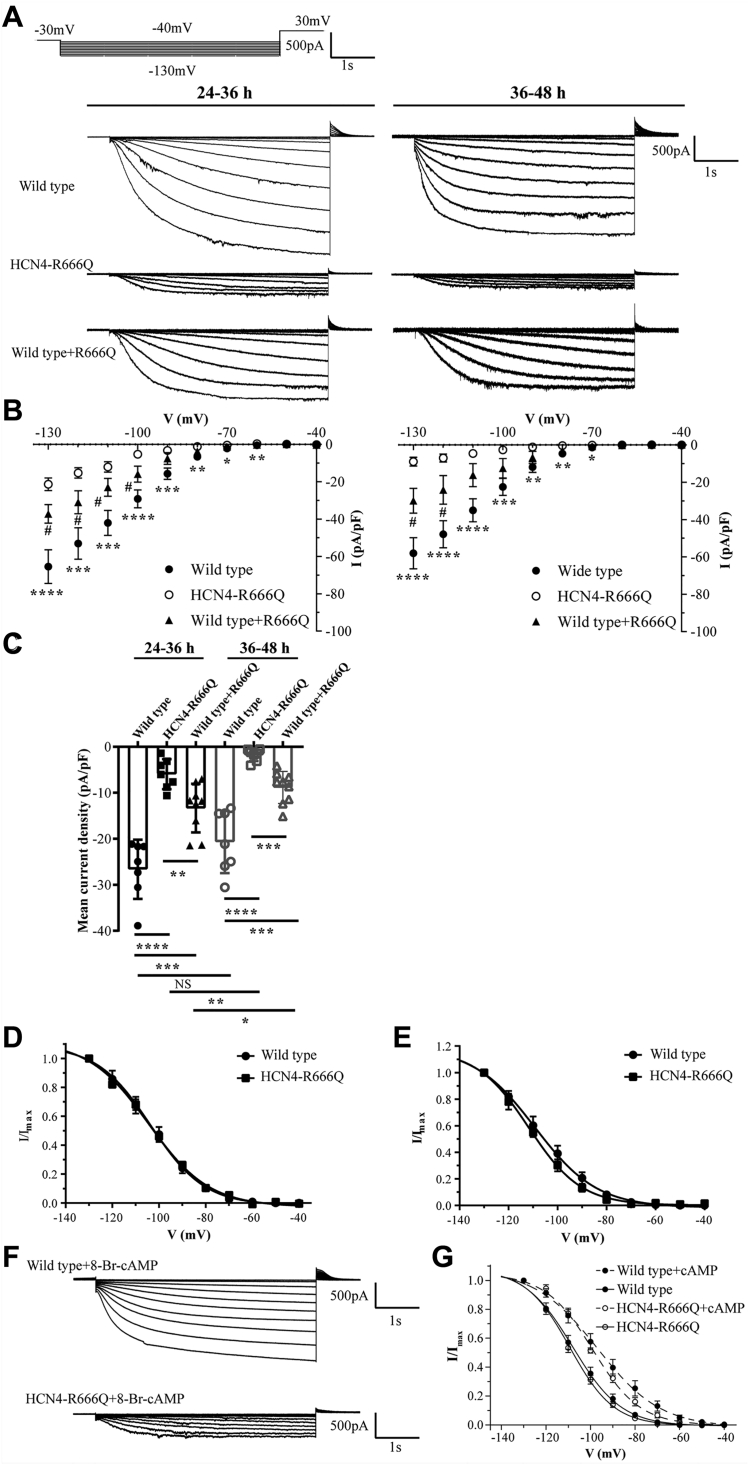
**Electrophysiological expression of wildtype or homomeric, heteromeric mutant channels in HEK293T cells**. *A*, currents recorded from cell transfected with either wildtype or homomeric, heteromeric mutant plasmid. The bath solution contained 75 mmol/L Na^+^ and 60 mmol/L K^+^. Voltage protocol (*Top*). Holding potential was -30 mV, and currents were elicited by 5 s hyperpolarizing voltage steps between −40 and −130 mV in 10 mV increments. *B*, the I-V relationship of wildtype (n = 7), homomeric mutant channels (n = 7), and heteromeric mutant channels (n = 9) in both 24 to 36 h (*left*) and 36 to 48 h (*right*) after transfection. *Asterisks* mark significant differences: Wildtype *versus* R666Q ∗*p* < 0.05, ∗∗*p* < 0.01, ∗∗∗*p* < 0.001, ∗∗∗∗*p* < 0.0001. Wildtype *versus* wildtype + R666Q ^#^*p* < 0.05, ^##^*p* < 0.01. *C*, mean current densities (±SD) at −130 mV generated by wildtype (n = 7), homomeric mutant channels (n = 7), and heteromeric mutant channels (n = 9) in 24 to 36 h and in 36 to 48 h after transfection. *D*, the activation curves of wildtype and homomeric mutant channels 24 to 36 h after transfection. *E*, the activation curves of wildtype and homomeric mutant channels 36 to 48 h after transfection. *F*, representative current traces of wildtype (n = 7), homomeric mutant channels (n = 7) in the presence of 1 mM 8-Br-cAMP. *G*, the activation curves of wildtype and homomeric and heteromeric mutant channels in the presence (*dashed line*) and absence (*solid line*) of 1 mM 8-Br-cAMP.

To investigate electrophysiological properties caused by the *HCN4*-R666Q mutation, we performed whole-cell voltage-clamp recordings of HEK293T cells transfected with only wildtype HCN4 or HCN4-R666Q (homomeric) or cells that were cotransfected with wildtype and HCN4-R666Q (heteromeric). A series of hyperpolarizing steps were applied to activate functional HCN4-mediated currents (Fig. 2*A*). The results showed that the amplitudes and density of wildtype HCN4 currents were significantly larger than the current amplitudes recorded from cells expressing mutant homomeric or heteromeric HCN4-R666Q channels in both 24 to 36 h and 36 to 48 h after transfection (Fig. 2, *A* and *B*). The mean current density of homomeric and heteromeric mutant channels and wildtype channels in 24 to 36 h and 36 to 48 h after transfection were also calculated. Interestingly, the mean current density of mutant channels was lower in 24 to 36 h after transfection and much lower in 36 to 48 h (Fig. 2*C*) compared to wildtype HCN4, respectively. The voltage for half maximal activation (V_1/2_), which was calculated by fitting the activation curve of HCN4 currents using the Boltzmann equation, was similar for both wildtype and homomeric HCN4- R666Q channels, suggesting that the HCN4-R666Q mutation did not influence channel activation kinetics (Fig. 2, *D* and *E* and Table S2). The voltage sensitivity of activation, k (slope of the simplified Boltzmann equation) also showed no significant difference between homomeric mutant and wildtype channels (Table S2).

### HCN4-R666Q mutation did not affect cAMP binding to the CNBD

Modulation of HCN4 channels is mediated by cAMP binding to the CNBD. Since residue R666 was located in the HCN4 CNBD region, we tested whether the *HCN4*-R666Q mutation affected cAMP binding to the CNBD. 1 mM the cAMP analog 8-Br-cAMP (Sigma-Aldrich) was included into patch pipettes. After breaking into the whole-cell mode, the cells 24 h after transfection were held for 10 min at a resting membrane potential of -30 mV to stabilize the intracellular 8-Br-cAMP concentration. The values of V_1/2_ of activation curves for wildtype and mutant HCN4-R666Q channels were shifted about 10 mV toward the depolarization direction (Fig. 2, *F* and *G*), whereas k values of the activation curves showed no significant difference between wildtype and homomeric HCN4-R666Q channels in the presence of 8-Br-cAMP (Table S3), indicating *HCN4*-R666Q mutation did not affect the cAMP dependence of channel activation. This was presumably due to HCN4-R666Q was located in the non-cAMP-binding region of HCN4 CNBD.

### Reduced protein levels of HCN4-R666Q mutant

**Figure 3:**
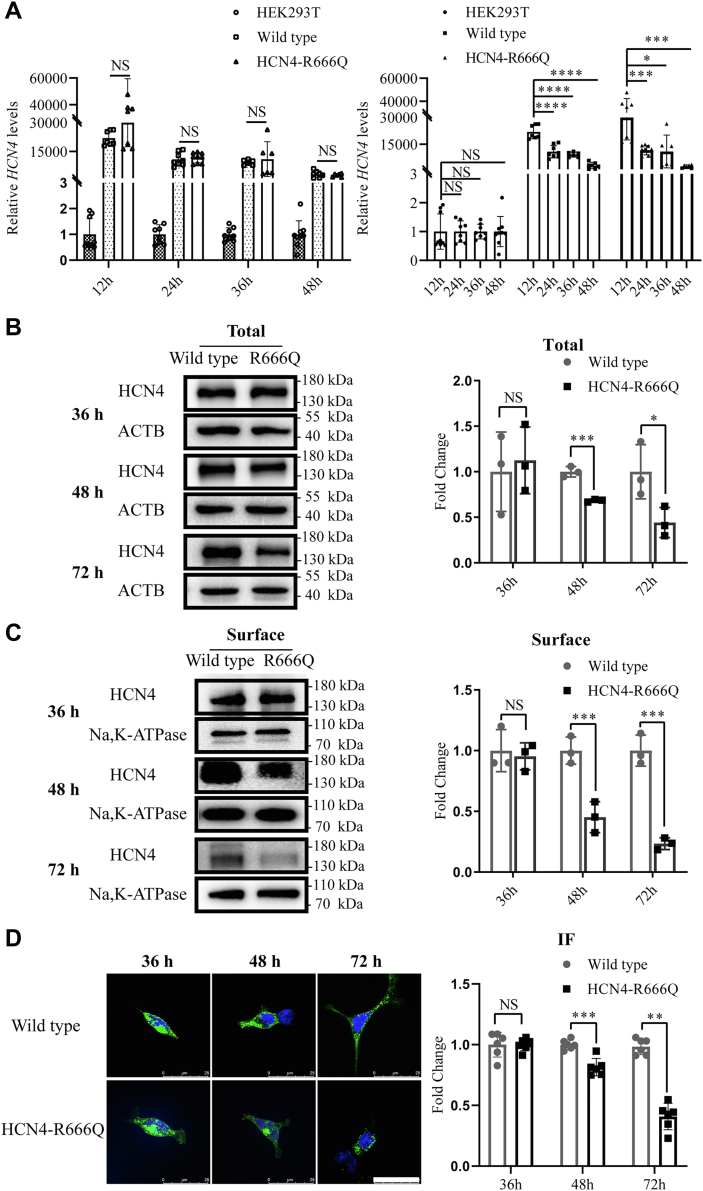
**The alteration of HCN4 expressional levels**. *A*, mRNA expression levels of wildtype (n = 6) and HCN4-R666Q (n = 6) mutant channels at 12 h, 24 h, 36 h, and 48 h posttransfection, respectively. NS indicates *p* > 0.05. *Right panel*, Comparison of decreased mRNA levels for both groups as time goes on. *B and C*, immunoblotting of total and surface protein using anti-HCN4 or anti-ACTB/anti-Na, K-ATPase of wildtype (n = 3) or HCN4-R666Q mutant (n = 3) plasmid transfected HEK293T cells at 36 h, 48 h, and 72 h posttransfection, respectively. *Right panel*, summary of the quantification of three independent experiments. *D*, confocal images of wildtype (n = 6) and HCN4-R666Q mutant (n = 6) protein expressed in HEK293T cells. The cells were stained with rat anti-HCN4 antibody (*green*) and DAPI (*blue*). Scale bar, 25 μm. *Right panel*, summary of the quantification of six independent cells. Asterisks mark significant difference: Wild type versus HCN4-R666Q ∗*p* < 0.05, ∗∗*p* < 0.01, ∗∗∗*p* < 0.001, ∗∗∗∗*p* < 0.0001.

Electrophysiological analysis showed that HCN4-R666Q channels reduced the current density more significantly in 36 to 48 h after transfection, so we determined whether this change was resulted from the alteration of mRNA or protein levels. There were no significant differences in the *HCN4* mRNA levels between HEK293T cells expressing only wildtype or HCN4-R666Q mutant channels at different time points (12, 24, 36, 48 h) after transfection (Fig. 3*A*). However, total protein and membrane protein levels at 48 h and 72 h after transfection were significantly decreased in HEK293T cells expressing homomeric HCN4-R666Q compared to those expressing wildtypeHCN4 (Fig. 3, *B* and *C*). The localization of the wildtype and homomeric HCN4-R666Q mutant protein by confocal microscopy also suggested that protein levels of HCN4-R666Q were decreased compared to wildtype (Fig. 3*D*).

### Increased degradation of HCN4-R666Q protein was regulated by ubiquitin-proteasome system

**Figure 4:**
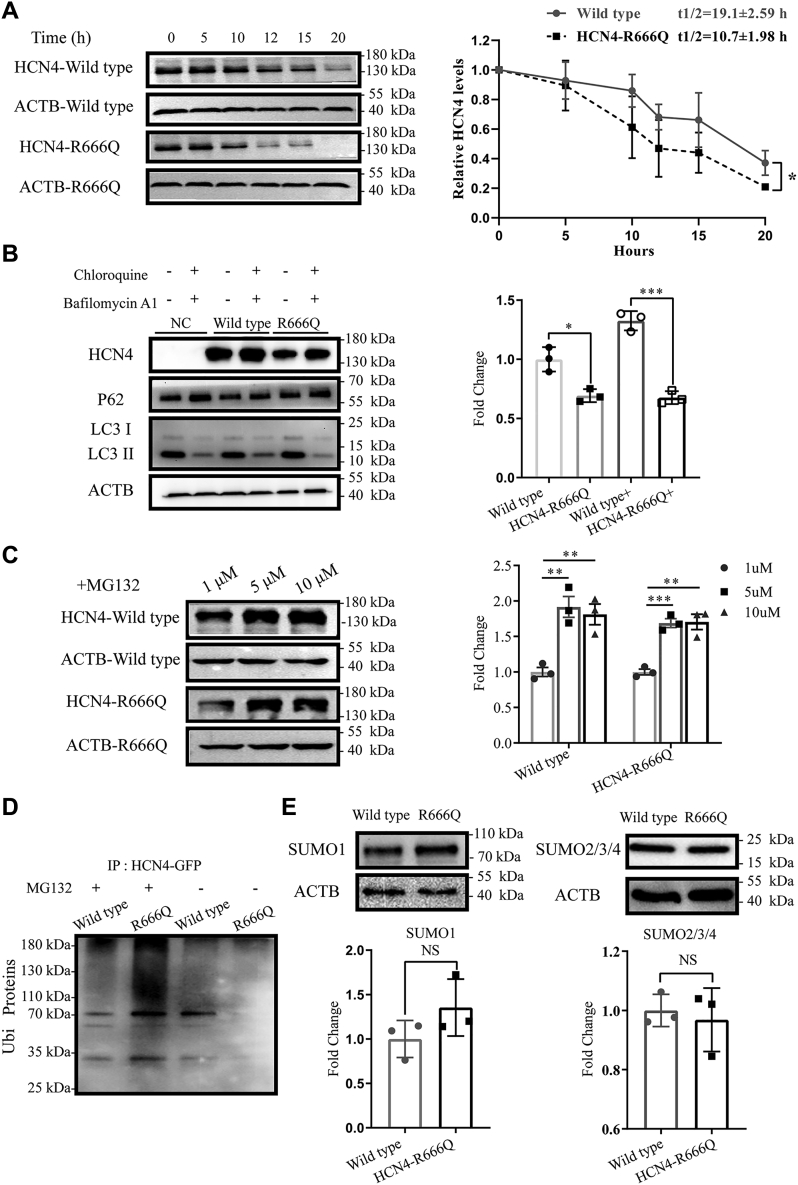
**The potential mechanism of the alteration of HCN4 mutant protein levels**. *A*, immunoblot analysis of wildtype and HCN4-R666Q mutant protein after treatment with CHX showed the reduction of HCN4-R666Q protein half-life, compared with wildtype. *Right panel*, the averages of three independent experiments are plotted. Error bars represent SD. Half-lives of the proteins were calculated by curve fitting. *B*, immunoblotting analysis indicated that HCN4-R666Q mutant increased the degradation of protein levels, which was not regulated by autophagy pathway. *Right panel*, summary of the quantification of three independent experiments. *C*, dose-dependent effect on proteasome inhibitor treatment. *Right panel*, summary of the quantification of three independent experiments. *D*, under the condition of protein denaturation, HCN4 protein was pulled down with GFP, then immunoblot analysis using ubiquitin antibody in HEK293T cells treatment with MG132. HCN4 was polyubiquitinated in MG132-treated cells. Untreated cells were used as controls. *E*, immunoblot analysis showed HCN4-R666Q mutant increased the degradation of protein levels, which was not regulated by SUMO1 (*Left*) and SUMO2/3/4 (*Right*). *Lower panel*, summary of the quantification of three independent experiments. Asterisks mark significant difference: Wild type versus HCN4-R666Q ∗*p* < 0.05, ∗∗*p* < 0.01, ∗∗∗*p* < 0.001. CHX, cycloheximide.

To understand the potential mechanism of the altered protein levels of HCN4-R666Q, we examined the half-life of wildtype and HCN4-R666Q protein. In HEK293T cells treated with the protein synthesis inhibitor cycloheximide (CHX) (31), the half-life of HCN4-R666Q mutant channel protein was significantly reduced compared with wildtype channels (19.1 ± 2.59 h *versus* 10.7 ± 1.98 h, *p* = 0.011; Fig. 4*A*), indicating the HCN4-R666Q protein was less stable and more rapidly degraded than wildtype.

Next, we investigated the pathway mediating HCN4-R666Q protein degradation. In transfected HEK293T cells treated with autophagy inhibitors, HCN4-R666Q protein levels were not rescued, although p62 and the ratio of LC3-I to LC3-II were accumulated (Fig. 4*B*). This result suggested that the autophagy pathway was successfully inhibited, and HCN4 mutant protein levels were not regulated by autophagy.

We next examined whether HCN4 protein were affected by the ubiquitin-proteasome system (UPS). MG132 affected HCN4 protein levels dose-dependently, and the optimal concentration was 5 μM (Fig. 4*C*). Furthermore, an immune-coprecipitation assay indicated that HCN4 was ubiquitinated, and ubiquitination levels of HCN4-R666Q mutant protein were higher than that of wildtype (Fig. 4*D*). Finally, we examined whether the SUMOylation, which was the reversible covalent attachment of a small ubiquitin-related modifier (SUMO) protein to a target protein, affected HCN4 protein levels. Immunoblot analysis showed that the expression levels of four SUMO isoforms (SUMO 1–4) were not significantly different between HCN4-R666Q mutant and wildtype (Fig. 4*E*). Taken together, these results indicated that the UPS pathway was considered as the potential mechanism of reduced HCN4-R666Q protein levels.

### Proteasome inhibitor treatment rescued reduction of HCN4-R666Q protein levels and current density

**Figure 5:**
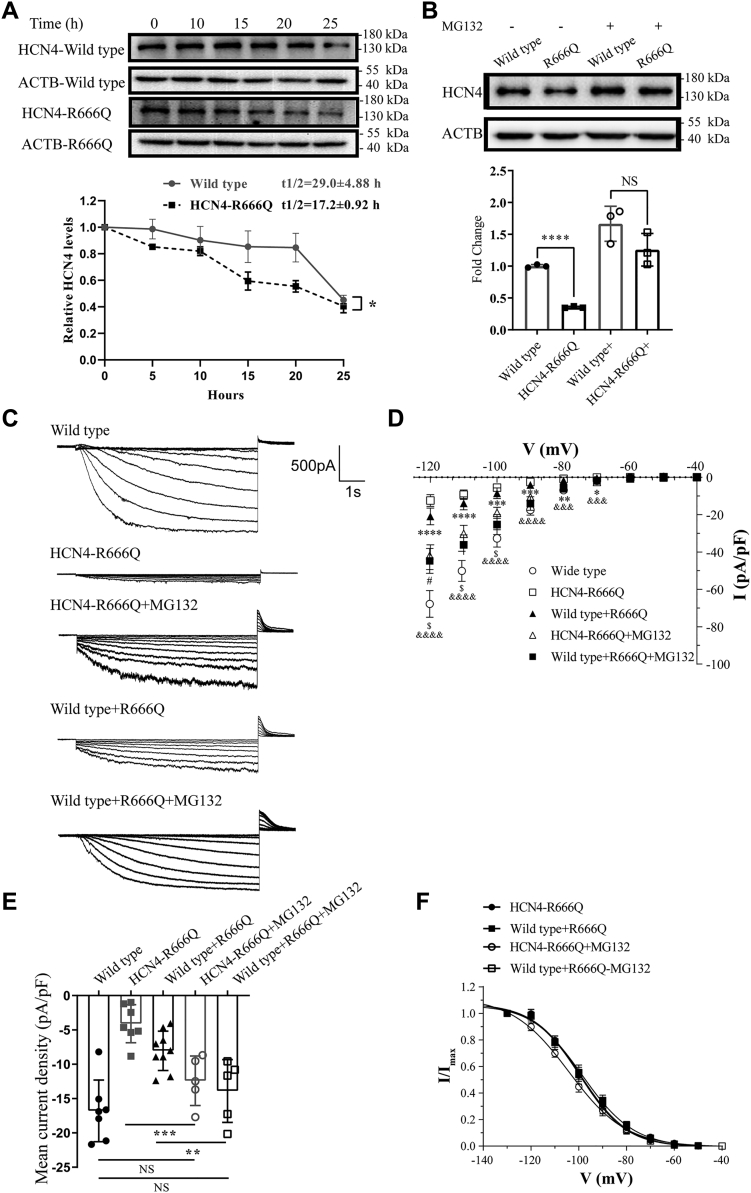
**Expression levels of wildtype and homomeric and heteromeric mutant channels in HEK293T cells in the absence and presence of proteasome inhibitor, respectively**. *A*, immunoblot analysis of HCN4 protein indicated that the HCN4-R666Q half-life was similar with wildtype after treatment with CHX and MG132. *Lower panel*, the averages of three independent experiments are plotted. Error bars represent SD. Half-lives of the proteins were calculated by curve fitting. *B*, immunoblot analysis of HCN4 protein showed MG132 rescued the HCN4 protein levels. *Lower panel*, summary of the quantification of three independent experiments. *C*, current traces recorded from cells transfected with either wildtype or homomeric or heteromeric mutant plasmid after treat with MG132. *D*, the I-V relationship of wildtype (n = 7), homomeric mutant channels (n = 7) and heteromeric mutant channels (n = 9) in the absence of 5 μM MG132 and homomeric mutant channels (n = 5) and heteromeric mutant channels (n = 5) in the presence of 5 μM MG132. Wildtype *versus* R666Q ^&^*p* < 0.05, ^&&^*p* < 0.01, ^&&&^*p* < 0.001, ^&&&&^*p* < 0.0001. Wildtype *versus* R666Q+MG132 ^$^*p* < 0.05. Wildtype *versus* wildtype+R666Q ∗*p* < 0.05, ∗∗*p* < 0.01, ∗∗∗*p* < 0.001, ∗∗∗∗*p* < 0.0001. Wildtype *versus* wildtype+R666Q+MG132 ^#^*p* < 0.05. *E*, the mean current density of wildtype (n = 7), homomeric mutant channels (n = 7), and heteromeric mutant channels (n = 9) in the absence of 5 μM MG132 and homomeric mutant channels (n = 5) and heteromeric mutant channels (n = 5) in the presence of 5 μM MG132. *F*, the activation curves of wildtype and homomeric and heteromeric mutant channels in the presence of 5 μM MG132. CHX, cycloheximide.

To explore the therapeutic potential of targeting *HCN4*-R666Q mutation, we determined the effect of proteasome inhibitor MG132 on rescuing the decreased protein levels and current density of HCN4-R666Q. HEK293T cells were transfected with wildtype or the homomeric HCN4-R666Q mutant and then treated with MG132 for 12 h. The half-life of the wildtype and HCN4-R666Q protein both were prolonged in the presence of MG132 (29.0 ± 4.88 h and 17.2 ± 0.92 h, respectively; *p* = 0.014) (Fig. 5*A*). Interestingly, in the presence of MG132 treatment, HCN4-R666Q protein levels were similar to that seen for wildtype in the absence of MG132 treatment (Fig. 5*B*). Consistently, decreased mutant channel current density was obviously rescued by treatment of transfected cells with MG132 for 12h (Fig. 5, *C*–*F*). However, there was no significant difference in V_1/2_ values after MG132 treatment, whereas the k was different apparently after MG132 treatment (Table S4).

## Discussion

**Figure 6:**
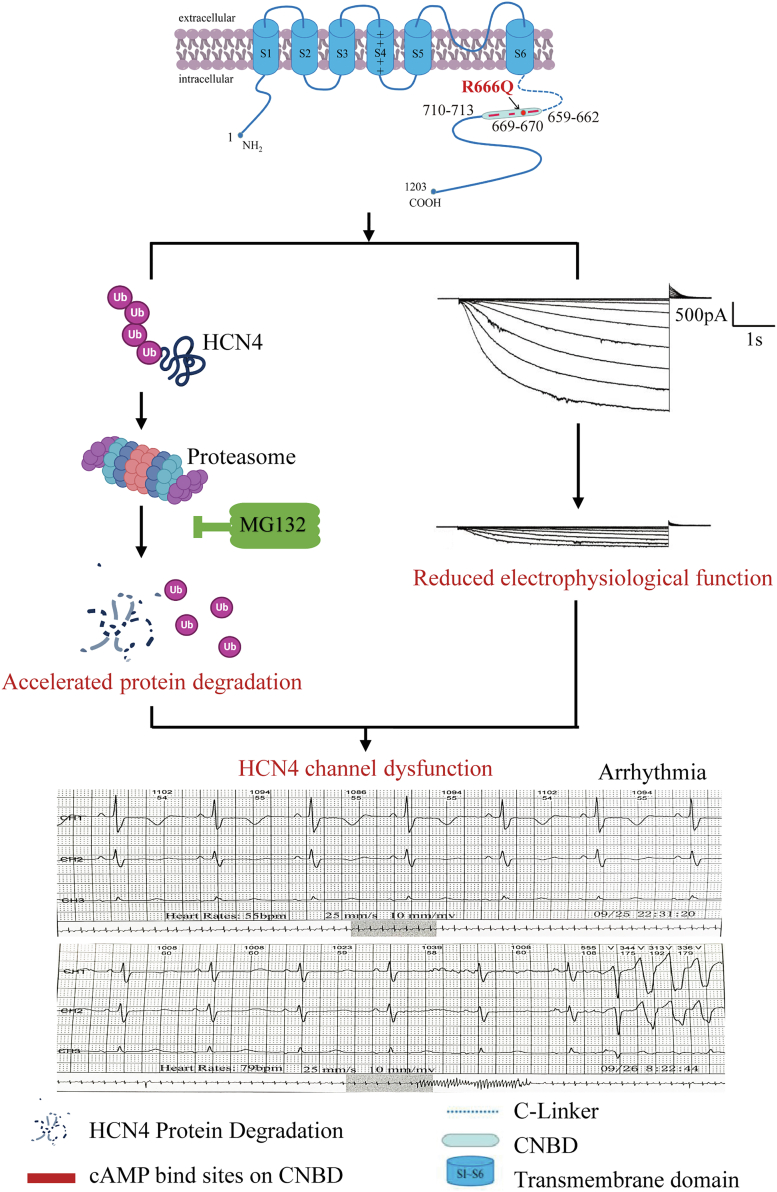
**Proposed model for *HCN4*-R666Q mutation leads to arrhythmias *via* ubiquitin-proteasome system was shown**. Our results revealed that the *HCN4*-R666Q mutation decreased the channel electrophysiological function (the image was from Fig. 2*A*) and increased its protein degradation, both resulted in reduced current density of HCN4 channels, accordingly may cause the occurrence of arrhythmia (the 12-lead ECGs was from patient B in Fig. 1*F*). We also found that proteasome inhibitor might has therapeutic benefits to mitigate the effects of this *HCN4* mutation. CNBD, cyclic nucleotide-binding domai; HCN, hyperpolarization-activated cyclic nucleotide-gatedn.

In the present study, we described the *HCN4*-R666Q mutation in two sporadic patients with sinus bradycardia, QT prolongation and short bursts of ventricular tachycardia. No other pathogenic mutations in the known arrhythmia-related genes were found in the two patients, suggesting that the loss of function of the *HCN4* mutation might be associated with sinus bradycardia, QT prolongation, and short bursts of ventricular tachycardia. We found the *HCN4*-R666Q mutation decreased the channel electrophysiological function and increased its protein degradation, both resulted in reduced current density of HCN4 channels. We also showed that proteasome inhibitor might has therapeutic benefits to mitigate the effects of this *HCN4* mutation (Fig. 6).

Two individuals in UK biobank (www.https://genebass.org/) carried *HCN4*-R666Q mutation, which has not been studied. The allele frequency of the mutation in the Non-Finnish European population was 3.72e-6. Interestingly, another mutation at residue 666, HCN4 p. Arg666Trp (R666W), appears to be associated with the susceptibility to atrial fibrillation (*p* = 9.7e-4). The allele frequency of *HCN4*-R666W in the Non-Finnish European population was 2.42e-5, indicating the significance of position 666 in HCN4. Several lines of evidences further suggested that the *HCN4*-R666Q mutation had functional consequences and was not simply a silent polymorphism. First, within the general population, the *HCN4*-R666Q variant was not found in the 1000 genomes, EPS6500, or the Exome Aggregation Consortium databases. Second, the mutation occurred in a segment that was highly conserved throughout evolution. Third, this substitution decreased electrophysiological function that underlies the clinical phenotype.

Nearly all reported *HCN4* mutations result in reduced current density of HCN4 channels that leads to slow heart rates. HCN4 channels are directly regulated by cAMP, which binds to CNBD of HCN4 channels and elicits a positive shift in the voltage dependence of activation. Notably, HCN4-R666Q had no dramatic impairments in cAMP activation in our study. Alternatively, the current reduction of HCN4-R666Q might be caused by a pronounced gating defect of HCN4-R666Q channels, which was independent of the cAMP modulation.

Aside from channel biophysical property, channel function was determined by the channel abundance at the plasma membrane. Our results revealed that mRNA levels were decreased for both groups as transfection time goes on. Previous studies have demonstrated that protein synthesis and degradation define protein turnover rates (32, 33). Immunoblotting and confocal microscopic analysis suggested that HCN4-R666Q led to an increase in the degradation of protein, suggesting that plasma membrane abundance was reduced. Similar decreasing protein levels was reported for *HCN4*-A485V mutation linked to symptomatic sinus bradycardia (17). Because surface expression of normal HCN4 channels was much decreased by the co-expression of mutant HCN4 channel, the *HCN4*-R666Q mutation serves as a dominant-negative suppression, which was also observed in previous studies on HCN4 channel loss of function mutations (1, 8, 9, 17, 19).

The decreased channel electrophysiological function and the markedly reduced plasma membrane protein as the major causes of the mutant HCN4 channel dysfunction. Our results showed that the loss of function in HCN4-R666Q resulted from the reduced surface expression rather than impaired cAMP modulation. Unlike the previously identified *HCN4*-P257S mutation (5), which reduced the expression of mutant channels due to defects in protein trafficking, decreased in *HCN4*-R666Q at the cell membrane appear to be due to degradation of protein. Protein degradation is one crucial component governing protein turnover (34). It is well established that the lysosomal and proteasomal systems are the two major protein degradation pathways in eukaryotic cells (35, 36, 37). Autophagy induces degradation of cytoplasmic materials and organelles in lysosomes, which plays an important role in maintaining cellular homeostasis (38, 39). The UPS plays an important role in the regulation of protein stability, which consists of ubiquitin-activating enzyme (E1), ubiquitin-conjugating enzyme (E2), and ubiquitin ligase (E3) that delivers ubiquitin from E2 to the specific substrates (40, 41). UPS pathway is involved in the turnover of multiple cellular proteins, enabling cells to dispose of biologically nonuseful proteins, including mutant, misfolded, and over accumulated proteins (42, 43). Our results revealed that increased degradation of HCN4-R666Q protein was regulated by the UPS rather autophagy pathway. The UPS, which is the major degradation pathway of intracellular proteins including voltage-gated channels (44, 45), is responsible for the degradation of most intracellular cytosolic, nuclear and membrane proteins, pivotal to both protein quality control and the regulatory degradation of normal proteins essential to virtually all cellular processes (46, 47, 48).

There is currently no targeted therapy for patients carrying *HCN4* mutations. Treatment of HEK 293T cells with the proteasome inhibitor MG132 partly rescued the decreased protein and current density of the mutant. Previous study has showed that HCN4 between cardiomyocytes and HEK293 cells exhibits a similar difference in V1/2 values, indicating that the factor contributing to the difference in voltage dependence affects both isoforms equivalently (49). Therefore, our discoveries can be translated to myocytes, which suggested that modulation of proteasome activity could be further explored as potential targeted therapy for patients with this mutation.

Cardiac ion channel dysfunction due to gene mutations displays a broad spectrum of clinical phenotypes (19). Known LQTS-causing mutations were not found in our study. Our results revealed *HCN4*-R666Q mutation in two sporadic patients with sinus bradycardia, QT prolongation, and short bursts of ventricular tachycardia. It is however important to note that, to our knowledge, the patients already described with QT prolongation due to HCN4 mutations has been reported were rare. Sinus bradycardia has been reported in relation to LQTS or HCN4 mutations. HCN channels play a role in slow diastolic depolarization during Phase 4 of cardiac action potential, especially in Purkinje cells and partly in sinus node cells, modulating heart rate produced by the primary or subsidiary pacemakers (17).

Proteasome activity is required for diverse cellular processes, including transcriptional and epigenetic regulation. MG132, which is a specific proteasome inhibitor, plays an essential role in inducing cell cycle arrest and apoptosis (50, 51, 52). Proteasome inhibition enhances antiproliferative while dampening cell-proliferative gene expression programs (53). Furthermore, MG132 has been determined to have anticancer effects in U2OS cells (54). In addition, proteasome inhibitors are promising antitumor drugs with preferable cytotoxicity in malignant cells and have exhibited clinical efficiency in several hematologic malignancies (55). It may also reduce the occurrence of ventricular arrhythmia such as heart failure (56). Our study revealed that MG132 may partly rescue the arrhythmia caused by *HCN4*-R666Q mutation. However, the tolerance and sensitivity of MG132 was different in different types of cells. MG132 is expected to affect many cellular processes, so targeted delivery is important for its therapeutic benefits.

Our study had some limitations. Firstly, we verified the abnormal expressional levels only in the HEK293T cell model transfected with HCN4-R666Q, it should be better if one more kind of cell models was used. Secondly, the patients were not followed-up due to the patients’ wishes, thus more clinical data were not received. Thirdly, the patch clamp experiment could only be performed within 48 h after transfection due to the current was unstable caused by poor cell status 72 h after transfection. Finally, HCN4 is the most abundant HCN isoform expressed in the human SA node and the atria. It would have been informative to determine if the *HCN4*-R666Q mutation segregated with QT prolongation, but the two patients refused our later follow-up.

In summary, the *HCN4*-R666Q mutation was found in two patients with sinus bradycardia, QT prolongation, and short bursts of ventricular tachycardia. This mutation resulted in a loss function of HCN4 channels that manifested as reduced *I*_f_ current by both decreased channel electrophysiological function and increased protein degradation. The reduced *I*_f_ current could be partly rescued by treatment with proteasome inhibitor. Our findings provided new insights into the pathogenesis of arrhythmias related to *HCN4* mutations.

## Experimental procedures

### Patients and clinical investigations

#### Existing Phenotype

In 2013 to 2016, two female patients (40 years; Patient A and 45 years; Patient B) suffering from palpitations and syncope were admitted to Fuwai Hospital as outpatients.

#### Family history

According to the two patients’ descriptions, both patients were normally healthy and had no medical history and familial cardiac events, sudden cardiac death, or other diseases and did not take drugs that could prolong QT.

#### Investigations

Patients were evaluated by clinical examination, 12-lead echocardiography, 24-h Holter monitoring, and Doppler echocardiography. The two patients’ parents and patient A’s son were evaluated by 12-lead ECG and Doppler echocardiography.

#### Follow-up

The patients were not followed-up in accordance with the patients’ wishes. The study was approved by the ethics committee of Fuwai Hospital, Peking Union Medical College (Approval No. 2012–400), Beijing, and conformed to the principles outlined in the Declaration of Helsinki. Written informed consent was obtained from each patient.

### Genetic analysis, in silico prediction algorithms, and mutagenesis

To determine whether the *HCN4*-R666Q mutation is pathogenic, we tested the pathogenicity potential based on *in silico* analyses, including Mutation Taster (http://www.mutationtaster.org/), PROVEAN (http://provean.jcvi.org/index.php), FATHMM (http://fathmm.biocompute.org.uk/), and PolyPhen-2 (http://genetics.bwh.harvard.edu/pph2/) (57, 58, 59).

To analyze the effect of the mutation in cells, we constructed plasmids with wildtype or mutated HCN4. Human *HCN4* cDNA (GenBank: NM_005477) was subcloned into GV230 vector containing a C terminally fused enhanced green fluorescent protein. Subsequently, the *HCN4*-R666Q mutation was introduced using the QuikChange II Site-Direct Mutagenesis Kit (Stratagene) into GV230 vector with appropriate mutagenic primers. All constructs were verified by Sanger sequencing.

### Cell culture and transfection

HEK293T cells were cultured in Dulbecco’s Modified Eagle’s Medium supplemented with 2 mM glutamine, 10% fetal bovine serum (Gibco), 1% P/S (10,000 Units/ml penicillin, and 10 mg/ml streptomycin) (Gibco) in 5% CO_2_ at 37 °C.

HEK293T cells, which were plated onto 35-mm gelatin-coated dishes, were transfected with 0.6 μg of wildtype or mutant HCN4-R666Q plasmid DNA using Lipofectamine 3000 (Invitrogen). In co-expression experiments, equal amounts (0.3 μg) of both plasmid DNAs (wildtype and HCN4-R666Q) were transfected. GFP-positive cells were used for whole-cell patch clamp experiments.

### Electrophysiological recordings

HCN4 channel-mediated currents were recorded in HEK293T cells 24 to 48 h after transfection using conventional whole-cell patch clamp techniques at room temperature (21–23 °C) as described (9, 11, 60). Signals were amplified using a HEKA EPC10 amplifier with the PatchMaster software (HEKA Instruments, Inc) Data were low-pass filtered at 2 kHz and acquired at 20 kHz. Patch electrodes had a resistance of 5 to 9 MΩ when filled with the pipette solution containing (in mmol/L) 115 KMeSO_4_, 1 NaCl, 0.1 CaCl_2_, 1 EGTA, 3 MgCl_2_ and 10 Hepes (pH 7.2 with KOH). The bath solution contained (in mmol/L): 60 KCl, 75 NaCl, 10 glucose, 2 CaCl_2_, 1 MgCl_2_, 1.2 NaH_2_PO_4_, and 10 Hepes (pH 7.3 with NaOH). Intracellular and extracellular solution osmolarity was adjusted with glucose to 290 and 300 mOsmol/L, respectively. Series resistances were electronically compensated by 60%. To evaluate functional properties, HCN4 currents were evoked by hyperpolarizing steps ranging from -40 to -130 mV (holding potential: -30 mV, increment: 10 mV, interval: 5 s). For drug application experiments, the transfected HEK293T cells were treated with 5 μM MG132 (a proteasome inhibitor) for 12 h, and current density was determined by dividing the fully activated current at -120 mV at the end of the 5s pulse by cell capacitance. The voltage dependence of channel activation was analyzed by fitting the Boltzmann function (I - I _min_)/(I _max_ - I _min_) = 1/(1 - exp [(V - V_1/2_)/k]), where V_1/2_ is the half-activation voltages, and k is the inverse slope factor.

### Expression analysis

At 12, 24, 36, and 48 h after transfection with wildtype or *HCN4*-R666Q, HEK293T cells were washed three times with ice-cold PBS, and total RNA was isolated using Trizol (Invitrogen). Then, 1 μg of total RNA was reverse transcribed using Hifair II 1st Strand cDNA Synthesis Super Mix. Real-time PCR assays were run in triplicate and quantified using the Prism 7500 sequence-detection system (ABI) with Hieff qPCR SYBR Green Master Mix. Relative mRNA expression was calculated by the ΔΔCt method, and the values were normalized to the expression of actin beta (ACTB). Primers used: *HCN4* forward 5′-TGGACACCGCTATCAAAGTGG-3′ and *HCN4* reverse 5′-CTGCCGAACATCCTTAGGGA-3′.

For immunoblotting, HEK293T cells at 36, 48, and 72 h after transfection were washed three times with ice-cold PBS, and total proteins were lysed with RIPA buffer and membrane proteins were extracted using a Minute Plasma Membrane Protein Isolation and Cell Fractionation Kit (Invent Biotechnologies). Protein samples were denatured by heating to 37 °C for 30 min and then used for immunoblot analyses. ACTB was served as a total protein control and Na, K-ATPase (CST, 1:1000) was used as a membrane protein control. All data shown represented at least three independent experiments.

### Immunofluorescence confocal microscopy analysis

Immunofluorescence assay was performed as described previously (8, 21). At 36, 48, and 72 h after transfection, HEK293T cells were grown on microscope slides and fixed in 4% preheated paraformaldehyde for 10 min. The fixed cells were rinsed three times with PBS, permeabilized with 0.1% Triton X-100 (Sigma-Aldrich) for 15 min, blocked for 1 h in 10% goat serum, and then incubated with HCN4 antibody (Abcam) overnight at 4 °C. The cells were then incubated with an Alexa Fluor 488-conjugated goat anti-rat antibody (Invitrogen) for 1 h in the dark and washed with PBS before DAPI staining and mounting in the dark. The cells were analyzed by fluorescence confocal microscopy (Leica). Identical parameters were used for image acquisition and analysis. A threshold was set for each image to eliminate background.

### Determination of HCN4 protein half-life

Protein half-life assay was performed as described previously (31). At 36 h after transfection, HEK293T cells were incubated with 25 μg/ml CHX (an inhibitor of protein synthesis) (61)for 12 h. Then, total protein was collected at different time points (0, 5, 10, 12, 15, and 20 h) for immunoblot analysis to monitor the HCN4 protein expression levels. Band intensities for immunoblotted HCN4 protein were quantified using the Image J software (National Institute of Health) and normalized to controls. The relative levels of HCN4 protein in sample not treated with CHX was considered as initial level which were referred to as 1 unit.

### Autophagy pathway assay

Autophagy pathway assay was performed as described previously (31, 62, 63, 64). At 36 h after transfection, HEK293T cells were treated with the autophagy inhibitors, bafilomycin A1 (50 nM) (MCE) and chloroquine (20 μM) (Sigma-Aldrich) for 24 h. Cell lysates in RIPA buffer were separated by 12% SDS-PAGE and then analyzed by immunoblotting using the following antibodies: HCN4 (1:5000; Abcam), p62 (1:10,000; Proteintech), LC3 (1:10,000; Proteintech) and ATCB (1:10,000; Proteintech).

### Ubiquitination and SUMO assays

Ubiquitination and SUMO assays were performed as described previously (31, 65). At 36 h after transfection, HEK293T cells were treated for 12 h with 1 μM, 5 μM, and 10 μM of the proteasome inhibitor MG132. In addition, HEK293T cells transfected with wildtype or HCN4-R666Q mutant plasmid were treated with 5 μM MG132 for 12 h. Total protein was collected and analyzed by immunoblotting using the following antibodies: SUMO1 (1:1000, Proteintech) and SUMO2/3/4 (1:1000, Santa Cruz).

### Co-immunoprecipitation analysis

Co-immunoprecipitations were performed as previously described (66, 67, 68). Briefly, HEK293T cells were grown on 10 cm dishes and 48 h after transfection, washed three times with ice-cold PBS, and lysed with ice-cold IP lysis buffer containing 1× protease inhibitors for 30 min on ice followed by ultrasonic lysing for 1 min. The cell lysates were incubated for 60 min and then centrifuged at 10,000*g* at 4 °C for 20 min. The lysates were precleared by incubation with protein A/G-agarose (Proteintech) for 1 h at 4 °C. Specific antibodies were incubated overnight at 4 °C followed by protein-A/G agarose beads incubation at 4 °C for 4 h. After the beads were washed three times with washing buffer, the immunoprecipitation was heated in 2× sample buffer at 37 °C for 30 min and then separated by SDS-PAGE. The separated proteins were analyzed *via* immunoblot.

### Statistical analysis

Data are reported as the mean ± SD. Statistical analysis of the results of >2 groups was carried out by one-way ANOVA followed by the least significant difference test or Tukey test. Student’s *t* test was performed for comparison of two groups only. *p* < 0.05 was considered statistically significant; 2-tailed tests were used for all analyses. Analysis of the Western blot bands was performed with ImageJ image analysis software. All measurements were obtained from distinct samples. Electrophysiological data were analyzed with patchmaster software. All statistical analyses were performed using GraphPad Prism 8.0.

## Data availability

Raw data will be available upon request.

## Supporting information

This article contains supporting information.

## Conflict of interests

The authors declare no conflicts of interest with the contents of the article.
