# Supplementary material for: Channel HCN4 mutation R666Q associated with sporadic arrhythmia decreases channel electrophysiological function and increases protein degradation
Source: J Biol Chem. 2022 Oct 14;298(11):102599. doi: 10.1016/j.jbc.2022.102599 (PMC9663530; doi:10.1016/j.jbc.2022.102599)
Supplement: Supplemental Information [file mmc1.docx]

**Supplemental Information**

**Figure Legends**

Figure S1. The 12-lead ECG data of two patients’ family members. A-C. 12-lead ECGs of patient A’s father, mother and son, respectively. D-E. 12-lead ECGs of patient B’ father and mother.


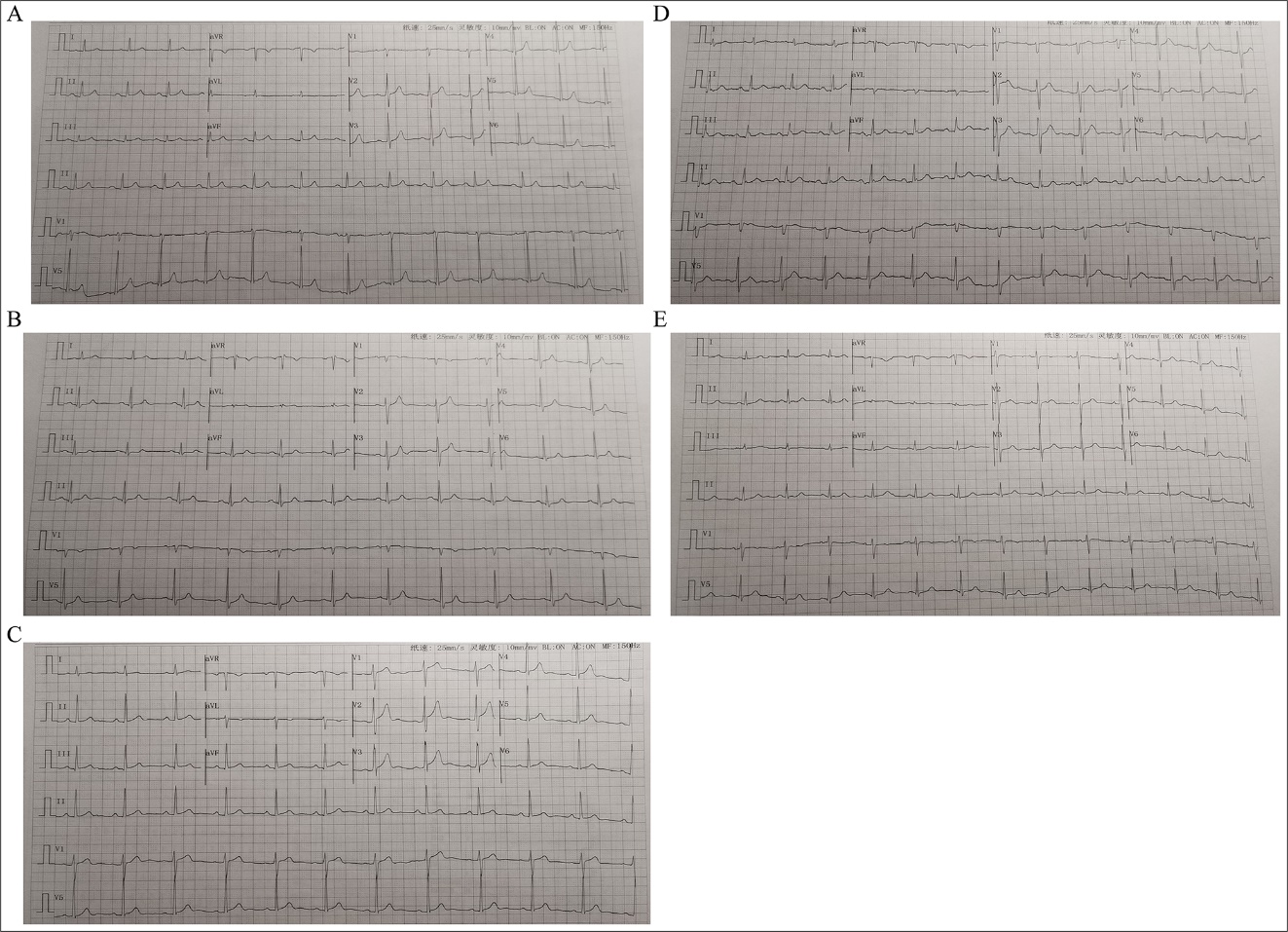


Figure S2. DNA sequencing results of two patients’ family members. A-C. DNA sequencing results of patient A’s father, mother and son, respectively. D-E. DNA sequencing results of patient B’ father and mother.


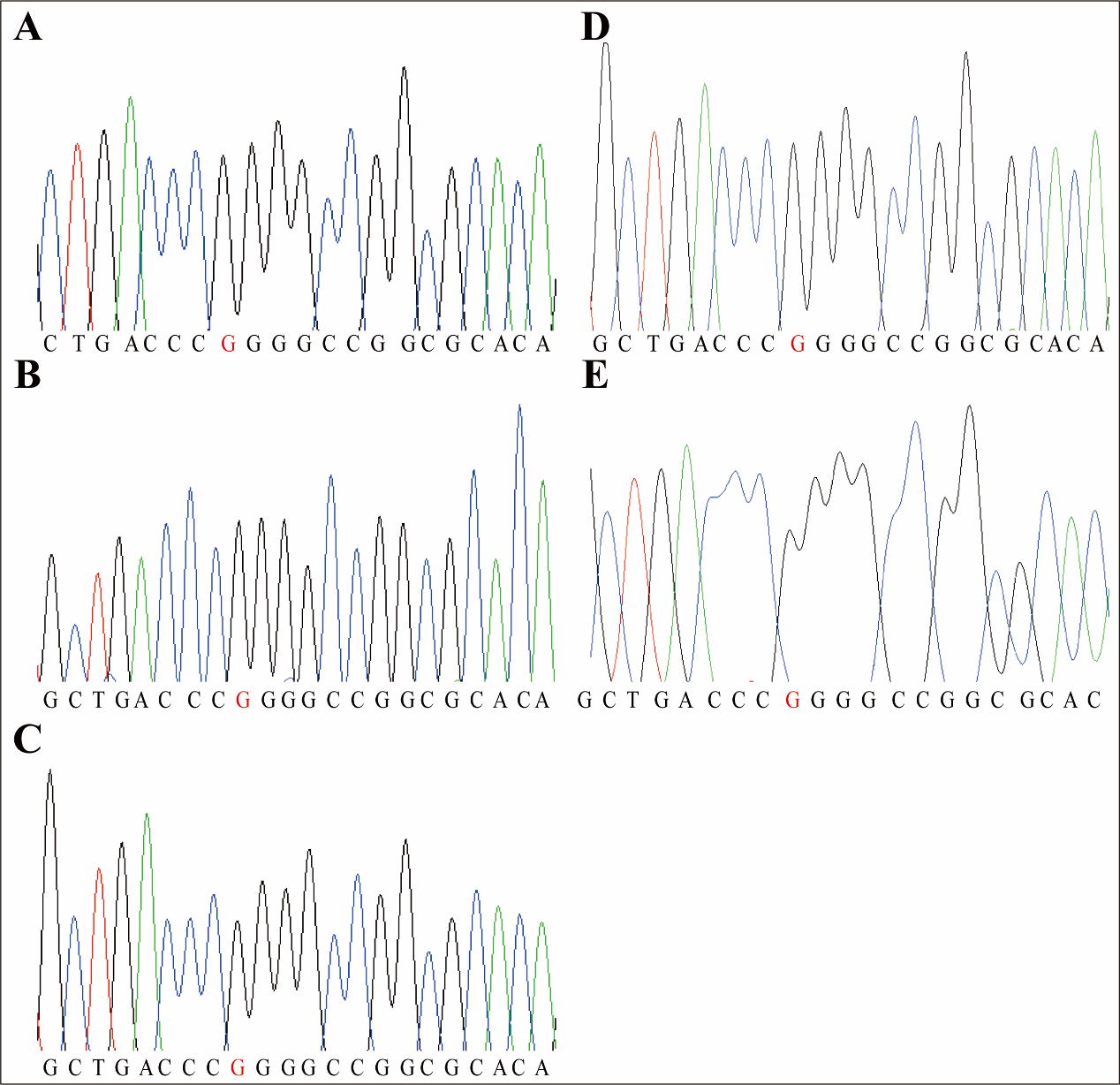


Table S1. The echocardiography data of two patients’ and their family members.

|  |  | Age (years) | AVAD (mm) | LAD (mm) | IVSD (mm) | LVEF (%) | LVEDD (mm) | LVEDV (ml/m^2^) | LVESD (mm) | LVESV (ml/m^2^) | LVPWD (mm) | LVSV (ml/m^2^) | RVD (mm) | RVMAPD (mm) |
| --- | --- | --- | --- | --- | --- | --- | --- | --- | --- | --- | --- | --- | --- | --- |
| 1-II.1 | Patient A | 40 | 18 | 36 | 10 | 76 | 39 | 66 | 22 | 16 | 10 | 50 | 31 | 24 |
| 1-I.2 | Patient A’ father | 73 | 22 | 36 | 9 | 63 | 49 | 113 | - | - | 10 | - | 19 | 23 |
| 1-I.1 | Patient A’ mother | 70 | 18 | 29 | 9 | 65 | 47 | 102 | 30 | 35 | 9 | 67 | 22 | 24 |
| 1-III.1 | Patient A’ son | 25 | 22 | 34 | 10 | 62 | 52 | - | - | - | 9 | - | 22 | 24 |
| 2-II.1 | Patient B | 45 | 23 | 33 | 9 | 62 | 45 | 92 | 30 | 35 | 10 | 57 | 21 | 22 |
| 2-I.2 | Patient B’ father | 75 | 23 | 29 | 10 | 60 | 44 | 88 | - | - | 10 | - | 21 | 18 |
| 2-II.1 | Patient B’ mother | 75 | - | 37 | 7 | 64 | 45 | 92 | - | - | 7 | - | 22 | 23 |

AVAD: Aortic annulus diameter; LAD: Left atrial diameter; IVSD: Intraventricular septum diastole; LVEF: Left ventricular ejection fraction; LVEDD: Left ventricular end diastolic diameter; LVEDV: Left ventricular end diastolic volume; LVESD: Left ventricular end systolic diameter; LVESV: Left ventricular end systolic volume; LVPWD : Left ventricular posterior wall thickness in diastole; LVSV:  left ventricular end-systolic volume; RVD: Right ventricular diameter; RVMPAD: Right ventricular monophasic action potentials durations

Table S2. Summary of the electrophysiological properties of whole-cell HCN4 channel currents.

|  | Wild type 24-36 h (A) | HCN4-R666Q24-36 h (B) | Wild type 36-48 h (C) | HCN4-R666Q 36-48 h (D) | *P* value (A) vs. (B) | *P* value (C) vs. (D) |
| --- | --- | --- | --- | --- | --- | --- |
|  | N=7 | N=7 | N=7 | N=7 |  |  |
| V_1/2_ | -103.6±1.993 | -104.4±1.517 | -109.2±3.068 | -111.7±2.234 | 0.414 | 0.107 |
| k | -11.59±1.604 | -12.93±1.206 | -12.69±2.028 | -10.68±1.426 | 0.102 | 0.053 |

Table S3. Summary of the electrophysiological properties of whole-cell HCN4 channel currents in the presence of cAMP.

|  | Wild type+ cAMP (A) | HCN4-R666Q+ cAMP (B) | *P* value (A) vs. (B) |
| --- | --- | --- | --- |
|  | N=7 | N=7 |  |
| V_1/2_ | -97.13±2.992 | -100.0±0.985 | 0.123 |
| k | -13.12±2.909 | -11.38±0.864 | 0.656 |

Table S4. Summary of the electrophysiological properties of whole-cell HCN4 channel currents in the presence of MG132.

|  | HCN4-R666Q (A) | Wild type+HCN4-R666Q (B) | *P* value (A) vs. (B) |
| --- | --- | --- | --- |
|  | N=5 | N=5 |  |
| V_1/2_ | -101.4±1.133 | -99.37±1.502 | 0.594 |
| k | -12.45±0.959 | -9.747±1.332 | 0.0009 |
